# Molecular Dynamic Analysis of Hyaluronic Acid and Phospholipid Interaction in Tribological Surgical Adjuvant Design for Osteoarthritis

**DOI:** 10.3390/molecules22091436

**Published:** 2017-09-04

**Authors:** Jacek Siódmiak, Piotr Bełdowski, Wayne K. Augé, Damian Ledziński, Sandra Śmigiel, Adam Gadomski

**Affiliations:** 1 Institute of Mathematics and Physics, UTP University of Science and Technology, 85-796 Bydgoszcz, Poland; jacek.siodmiak@utp.edu.pl (J.S.); piotr.beldowski@utp.edu.pl (P.B.); adam.gadomski@utp.edu.pl (A.G.); 2 Department of Research and Development, NuOrtho Surgical, Inc., Boston, MA 02723, USA; nnmoc@aol.com; 3 Faculty of Telecommunications, Computer Science and Technology, UTP University of Science and Technology, 85-796 Bydgoszcz, Poland; damian.ledzinski@utp.edu.pl; 4 Faculty of Mechanical Engineering, UTP University of Science and Technology, 85-796 Bydgoszcz, Poland

**Keywords:** osteoarthritis, hyaluronic acid, phospholipids, molecular dynamics simulation

## Abstract

Tribological surgical adjuvants constitute a therapeutic discipline made possible by surgical advances in the treatment of damaged articular cartilage beyond palliative care. The purpose of this study is to analyze interactions between hyaluronic acid and phospholipid molecules, and the formation of geometric forms, that play a role in the facilitated lubrication of synovial joint organ systems. The analysis includes an evaluation of the pathologic state to detail conditions that may be encountered by adjuvants during surgical convalescence. The synovial fluid changes in pH, hyaluronic acid polydispersity, and phospholipid concentration associated with osteoarthritis are presented as features that influence the lubricating properties of adjuvant candidates. Molecular dynamic simulation studies are presented, and the Rouse model is deployed, to rationalize low molecular weight hyaluronic acid behavior in an osteoarthritic environment of increased pH and phospholipid concentration. The results indicate that the hyaluronic acid radius of gyration time evolution is both pH- and phospholipid concentration-dependent. Specifically, dipalmitoylphosphatidylcholine induces hydrophobic interactions in the system, causing low molecular weight hyaluronic acid to shrink and at high concentration be absorbed into phospholipid vesicles. Low molecular weight hyaluronic acid appears to be insufficient for use as a tribological surgical adjuvant because an increased pH and phospholipid concentration induces decreased crosslinking that prevents the formation of supramolecular lubricating forms. Dipalmitoylphosphatidylcholine remains an adjuvant candidate for certain clinical situations. The need to reconcile osteoarthritic phenotypes is a prerequisite that should serve as a framework for future adjuvant design and subsequent tribological testing.

## 1. Introduction

Synovial joint organ system lubrication is a complex process influenced by mechanical, molecular, and fluid factors toward wear reduction and tissue homeostasis. In healthy systems, an effective lubricating molecular layer is formed between juxtaposed articular cartilage surfaces. Both structural and compositional changes in this layer can lead to aberrant lubrication, ineffective perturbation mitigation, and progressive system dysfunction. Because surgical advances have enabled the non-palliative repair of damaged cartilage surfaces without producing iatrogenic collateral tissue necrosis, an interest in normalizing this molecular lubricating layer has emerged as the therapeutic discipline of tribological surgical adjuvants [1]. Yet, a confounding question lingers: can or should this molecular layer be normalized to enhance tissue rescue while pathologic phenotypes may persist post-treatment [2]? Though knowledge of osteoarthritis’ phenotype spectrum appears ever increasing [3], this study is designed as a framework to evaluate synovial fluid conditions that adjuvant candidates may encounter during surgical convalescence.

**Figure 1:**
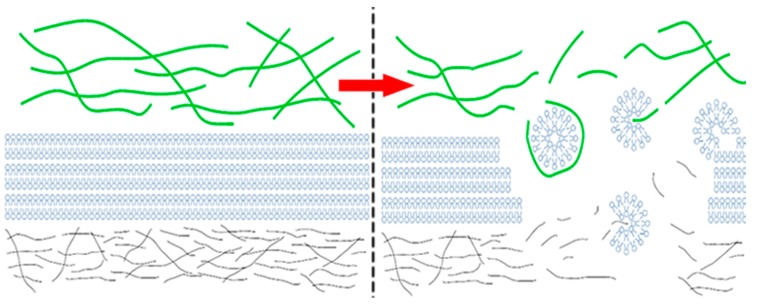
Graphical simplification of a synovial joint organ system. The left-hand side depicts normal synovial fluid conditions with longer hyaluronic acid (HA) chains and mature HA networks (green), phospholipid (PL) bilayers of the oligolamellar surface-active phospholipid layer (blue), and the superficial zone of articular cartilage (grey). The right-hand side depicts osteoarthritic synovial fluid conditions with correspondingly shorter HA chains, rudimentary HA networks, PL micelles, and a damaged superficial zone upon which the surface-active phospholipid layer cannot form.

**Figure 2:**
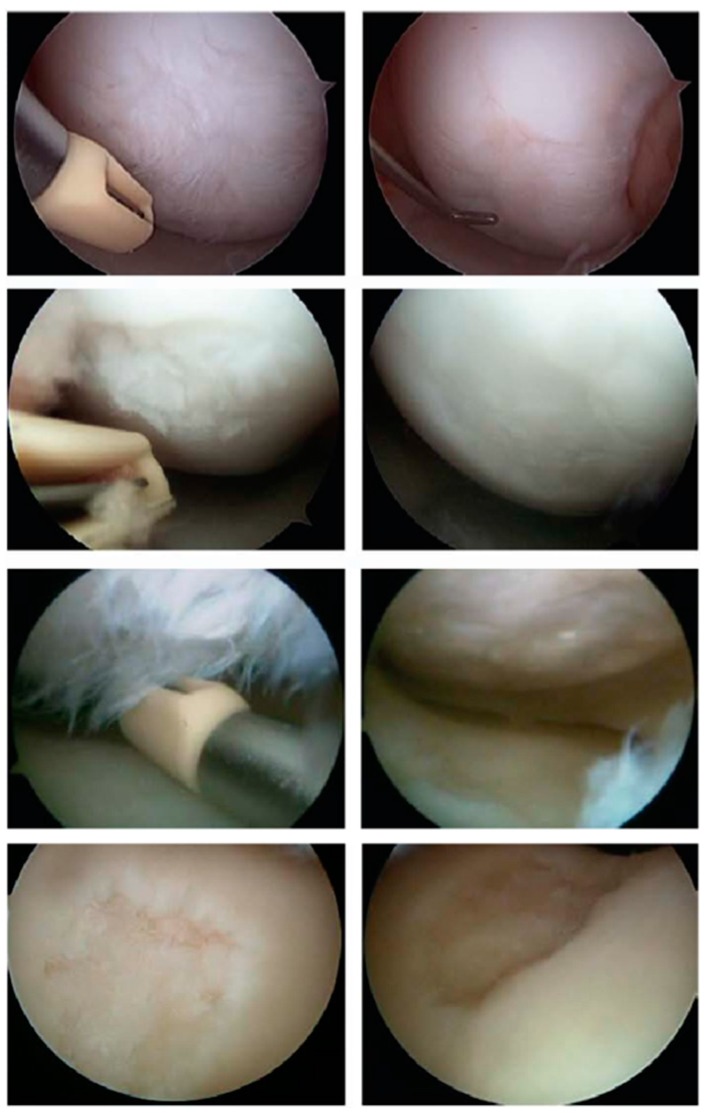
Pre-treatment (**Left**) and post-treatment (**Right**) images of human knee articular cartilage surfaces. The surgical device shown is a physiochemical scalpel designed to repair damaged biologic tissue surfaces by mimicking the respiratory burst myeloperoxidase system of azurophilic granules in polymorphonuclear neutrophil granulocytes that deliver protonation potentials during the acute phases of wound healing. Image reproduced with permission from [4].

Hyaluronic acid (HA) and phospholipids (PL) are key constituents of the lubricating molecular layer in synovial joint organ systems. In addition, due to wide biocompatibility profiles and many established therapeutic uses, these molecules have been considered important candidates for tribological surgical adjuvant design [1]. Both have been used clinically for short-term palliative relief, but have been ineffective as tribological agents when administered without surgical lesion repair first. As depicted in Figure 1 and Figure 2, changes in these molecules occur commensurate with the degree of surface damage associated with system disease. Because damaged articular cartilage cannot support the formation of this lubricating molecular layer, these molecules form divergent complexes at and around the damage site rather than effective tribological structures. Fortunately, surgical repair has enabled the ability to create a suitable substrate upon which a surface-active phospholipid layer can reconstitute [4,5,6,7]. However, the durability of this layer, and likewise the fate of surgical adjuvants delivered to improve the nature of this layer, remain at the mercy of osteoarthritic phenotypes which may persist and yield competing intra-articular processes. For this reason, tribological surgical adjuvant design must reconcile post-delivery system conditions.

Synovial fluid pH changes have been used to characterize relative degrees of inflammatory and non-inflammatory pathogenesis as an early effort toward treatment palliation [8,9,10]; nevertheless, pH-dependent changes in the interaction between HA and PL have been afforded little clinical attention despite the profound impact on overall tribological function. In osteoarthritis, synovial fluid pH tends toward higher ranges [11]. Addtionally, at the same time, HA concentration decreases and PL concentration increases when compared to normal synovial fluid profiles [12]. More importantly, however, is that the species profiles within these molecular classes change qualitatively and quantitatively. For instance, HA chain length polydispersity is altered toward smaller-sized chains which display dissimilar intra- and inter-chain crosslinking affinities [13]; and, PL composition is significantly transformed [14] toward molecular saturation levels and fatty acid chain lengths less characteristic of those suited for synovial joint organ system lubrication [14,15].

Previous model system observations indicate that lubrication-related molecules can be both chemically and physically attached to surfaces such that chemically grafted constructs provide better wear protection and show a decrease in frictional coefficients than do constructs attached only by physical mechanisms [16]. Such observations support current in vivo findings that to produce surfaces which display durable wear properties, damaged articular cartilage requires *physiochemical* surgical repair [4,5,6,7]. Recognizing that the interfacial functional dynamics of the superficial active phospholipid layer display features of a sacrificial layer in dissipating perturbation forces and that favorable absorption–desorption kinetics are required to preserve that function [4,7], intra-articular conditions post-delivery are important to study before exploring the tribological effects of various adjuvant preparations in limiting articular cartilage wear [17,18].

In osteoarthritis, the total concentration of hyaluronic acid is slightly changed, but not all hyaluronic acid molecules are present in a long form due to weight distribution changes. The purpose of the present research is to investigate how the short faction of HA molecules behave in the event of an increased phospholipid concentration characteristic of osteoarthritis and to demonstrate that short HA molecular chains, by the interactions with PL, do not participate in the crosslinking of long HA molecular chains. This study examines the influence of pH on low molecular weight HA (50 nm; 20 kDa) and interactions amongst dipalmitoylphosphatidylcholine (DPPC) molecules, with pH = 7 representing healthy synovial fluid and pH = 8 representing osteoarthritic pathogenesis. Synovial fluid HA molecular weight ranges from ~4 kDa to ~8 MDa, wherein higher molecular weight species, which are selectively lost commensurate with osteoarthritis [19,20], are associated with lubricating properties. Thus, examining low molecular weight HA is deemed to represent an osteoarthritic phenotype expected to display suboptimal features as an adjuvant candidate. DPPC is a saturated zwitterionic surfactant normally present in synovial joint organ systems at ~8–11% of total phosphatidylcholine species and which has been used clinically to treat osteoarthritis [15].

Three DPPC concentrations are examined based upon normal concentration values (HA ~2.2 mg/mL; DPPC ~314.2 nmol/mL) expressed as a mass ratio of PL to HA, with $k_{PL:HA}=1:8$ corresponding to healthy synovial joint organ systems, $k_{PL:HA}=1:5$ to early osteoarthritis, and $k_{PL:HA}=2:7$ to advanced osteoarthritis. The radius of gyration (*R_g_*) is evaluated to reflect relationships between protein size, folding rate, and spatial structure [21,22,23]; root-mean-square deviation (RMSD), to analyze atomic position; and mean squared displacement (MSD), to assess the deviation time between molecular positions in an aqueous sodium chloride environment without other molecules. The Rouse model is applied to end-to-end vector analysis to evaluate polymer chain spread in space; and molecular surface area availability is evaluated with hydrophobicity and hydrogen bond number and their corresponding bond energies.

## 2. Results

### 2.1. Radius of Gyration: Time Evolution; pH Value; and PL Concentration Dependence

**Figure 3:**
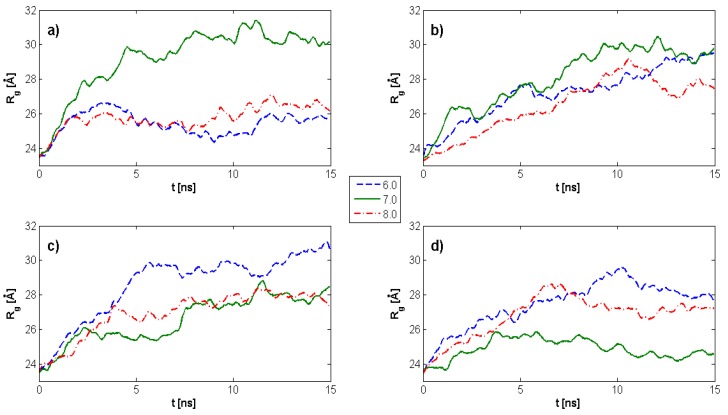
Time evolution of the HA radius of gyration as a function of pH values and dipalmitoylphosphatidylcholine (DPPC) concentration. (**a**) $k_{PL:HA}=0$; (**b**) $k_{PL:HA}=1:8$; (**c**) $k_{PL:HA}=1:5$; (**d**) $k_{PL:HA}=2:7$. The legend indicates pH values.

Figure 3 demonstrates the time evolution of HA *R_g_* as a function of pH values and DPPC concentration. The maximum *R_g_* value for HA alone is observed for pH = 7. Further, *R_g_* is highly dependent on DPPC concentration, such that for all DPPC concentrations and pH values, *R_g_* tends toward a constant value. With an increasing DPPC concentration, *R_g_* progressively decreases over the ranges evaluated at pH = 7, which is quite distinct from the results at pH = 6 and pH = 8. In instances where DPPC is not present, a plateau begins to develop after 3–4 ns; while in the presence of DPPC, a similar plateau is observed only after 6–10 ns. This overall tendency toward a constant value can allow for a more general estimation of average *R_g_* values as a function of DPPC concentration within the plateau regions. The overall tendency is an increasing DPPC concentration, which causes HA to remain in an unfolded state longer, favoring crosslinking, and, in turn, facilitating lubrication.

**Figure 4:**
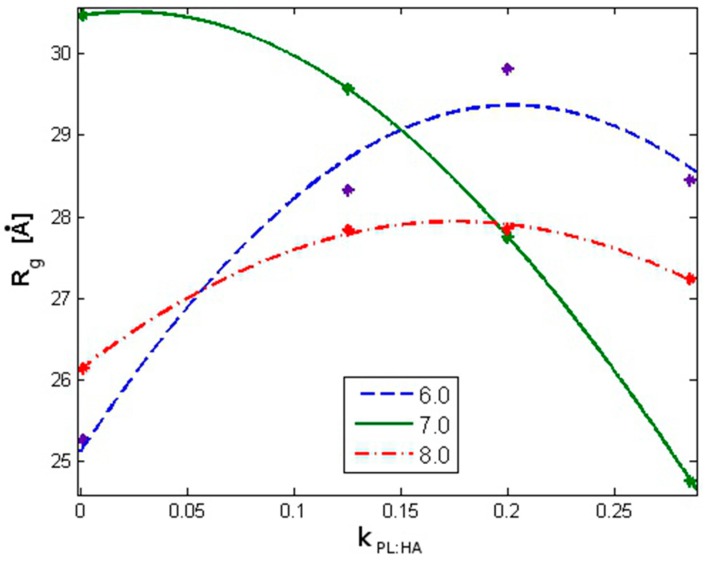
The average HA radius of gyration as a function of DPPC concentration defined as the mass ratio $k_{PL:HA}=\frac{PL}{HA}$. The lines represent Gaussian function fitting to mean values (represented by stars). The fitting parameters are presented in Table 1. The legend indicates pH values.

As shown in Figure 4 and Table 1, for pH = 7 and with an increasing DPPC concentration, the average *R_g_* decreases by approximately 20%; for pH = 6, the trend is reversed such that *R_g_* increases by approximately 20%. When pH = 8, only a weak dependence of *R_g_* on DPPC concentration is observed. The high *R*^2^ value (i.e., >0.9) indicates the model fits the experimental data well.

**Figure 5:**
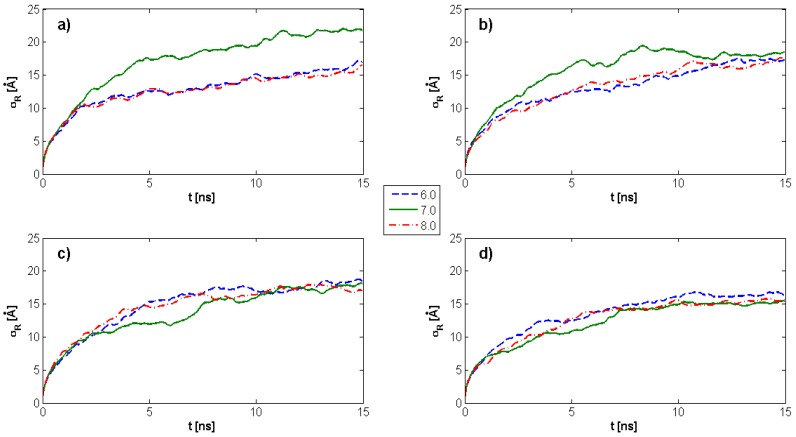
Root-mean-square deviation (RMSD) as a function of time for different pH values and DPPC concentrations. (**a**) $k_{PL:HA}=0$; (**b**) $k_{PL:HA}=1:8$; (**c**) $k_{PL:HA}=1:5$; (**d**) $k_{PL:HA}=2:7$. The legend indicates pH values.

The *R_g_* results are corroborated by analyzing the RMSD of atomic positions ($\sigma_{R}$) as a measure of the average distance between atoms that constitute a biopolymeric chain. As presented in Figure 5, RMSD also tends in time to a constant value; but, in contrast to the characteristics presented in Figure 3, these values are less perturbed.

**Figure 6:**
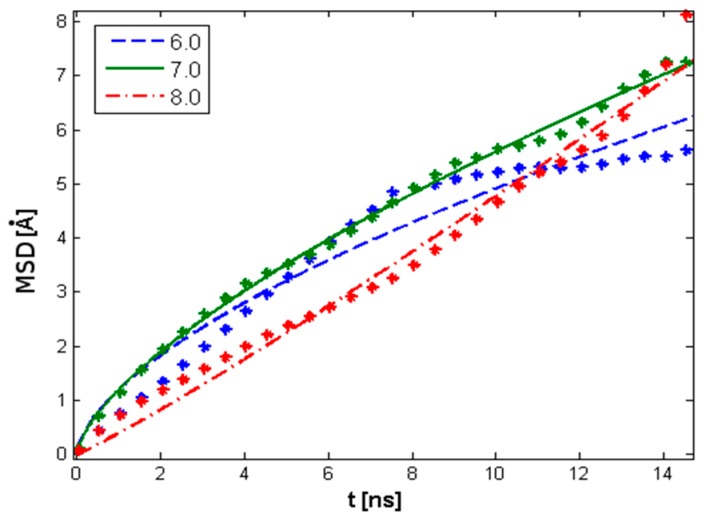
Mean square displacement (MSD) of single HA molecules at different pH values. The data has been fit using the exponential function $MSD\propto t^{\beta }$ such that when β=1, normal diffusion is represented; when β<1, subdiffusion is represented; and when β>1, superdiffusion is represented. Individual exponents are presented in Table 2. The legend indicates pH values.

Because the system contains interacting molecules which are immersed in a water environment, the statistical mechanics mean MSD measures the spatial extent of random motion, characterized as the amount of the system “explored” by a random walker. In this instance, a single HA molecule at different pH values can be analyzed as a further corroborative assessment. The ability to move is determined by the length and spread of the HA chain, the amount of phospholipid present, and the PL–HA interaction strength as a pH-dependent parameter. As presented in Figure 6 and Table 2, the MSD for pH = 6 and pH = 7 indicates subdiffusion behavior wherein movement is strongly constrained. For pH = 8, substantially normal diffusion is observed.

**Figure 7:**
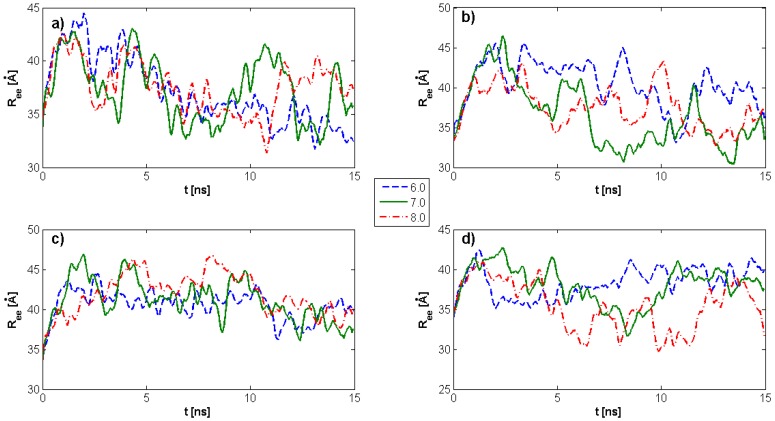
The HA end-to-end vector time evolution for different pH values and DPPC concentrations. (**a**) $k_{PL:HA}=0$; (**b**) $k_{PL:HA}=1:8$; (**c**) $k_{PL:HA}=1:5$; (**d**) $k_{PL:HA}=2:7$. The legend indicates pH values.

The end-to-end vector in polymer physical chemistry is the trajectory from one end of a polymer to the other and is a measure of the spread in space of the polymer chain. Figure 7 depicts the HA end-to-end vector time evolution for different pH values and DPPC concentrations. By employing the Rouse model to describe this variable, the stochastic process governing *R_ee_* behavior can be analyzed as an antecedent to an autocorrelation function.

**Figure 8:**
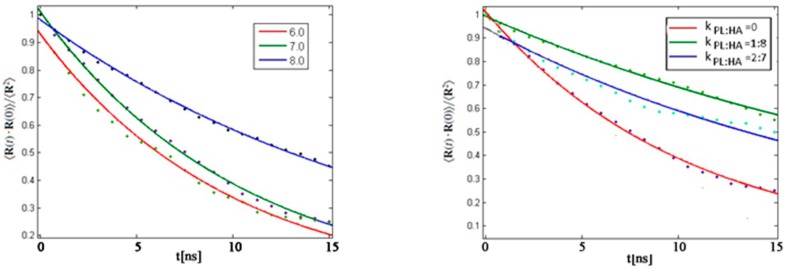
Autocorrelation function of the HA end-to-end vector. (**Left**) HA without DPPC for different pH values; (**Right**) HA with DPPC for pH = 7. The fit has been made using Equation (7). The legend indicates pH values.

In Figure 8, the autocorrelation function of the HA end-to-end vector is presented. The curve slopes describe the level of process randomness such that steeper curves depict a more random process. When DPPC is not present, as in Figure 8 Left, for pH = 6 and pH = 7, the autocorrelation function exhibits a higher slope, and consequently more randomness, than for pH = 8. Also recognized is that with the addition of DPPC, Figure 8 Right, HA behaves in a less random fashion and is governed by hydrophobic interactions between molecular species.

### 2.2. Surface Visualization: Surface Area Accessibility, Hydrophobicity, and Hydrogen Bonds

**Figure 9:**
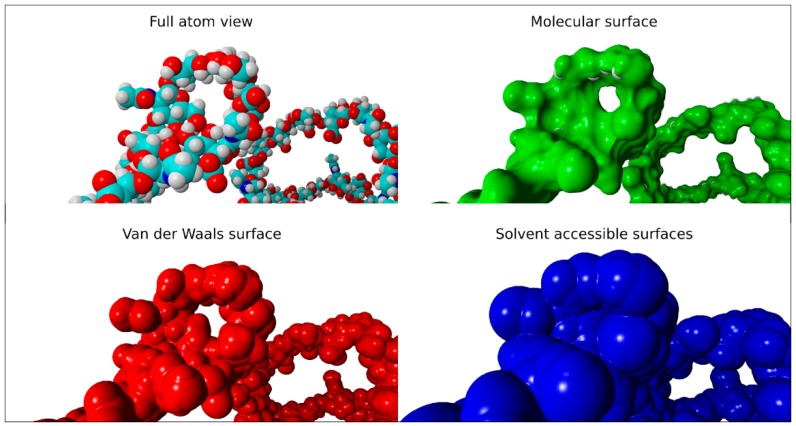
Full atom view of the hyaluronic acid molecule. The radius of water probe equals 1.4 Å. Red depicts oxygen; cyan, carbon; white, hydrogen; and blue, nitrogen.

Figure 9 depicts the molecular, van der Waals, and solvent-accessible surfaces of HA without the presence of PL molecules. Note the open structure consistent with larger *R_g_* values.

**Figure 10:**
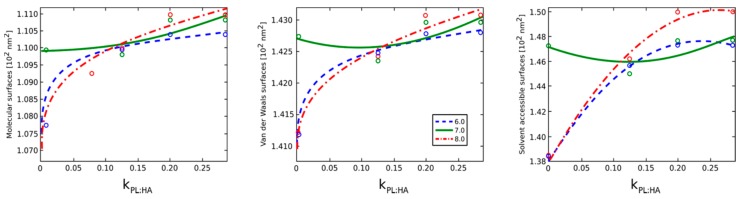
Molecular, van der Waals, and solvent-accessible surfaces as a function of pH values and DPPC concentration. The legend indicates pH values.

Figure 10 presents respective surface area accessibility changes as a function of pH value and DPPC concentration. For pH = 7.0, note that no significant changes are observed between DPPC concentrations when compared to other pH values that exhibit variable surface areas. The constant value of the surface area at pH = 7.0 is a result of the hydrophilic interactions between HA and DPPC molecules, such as from HA residue self-avoidance and water penetration into the PL–HA complex.

**Figure 11:**
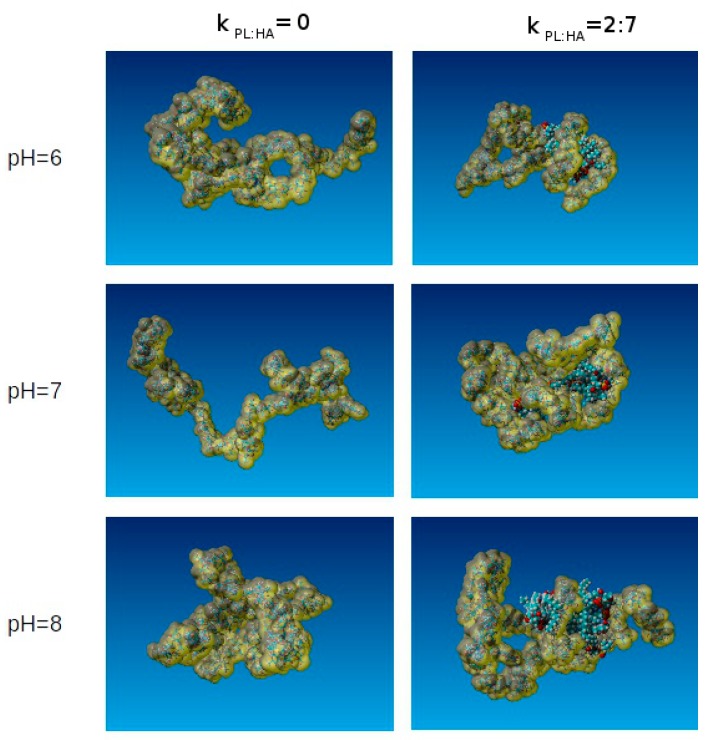
Visualization of solvent accessible surfaces as a function of pH and DPPC concentration. Red depicts oxygen; cyan, carbon; white, hydrogen; blue, nitrogen; and yellow, phosphorus.

Figure 11 depicts only the HA solvent-accessible surface structure as a function of pH values and DPPC concentration. Note the dramatic change at pH = 6 and pH = 8.

**Figure 12:**
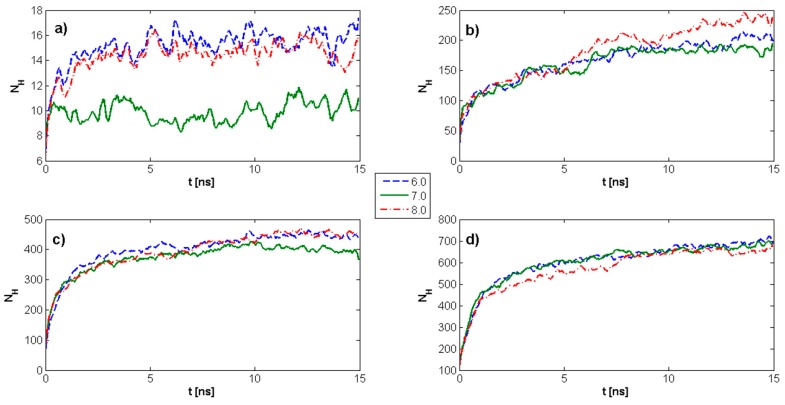
Number of hydrophobic interactions as a function of time for different pH values and DPPC concentrations. (**a**) $k_{PL:HA}=0$; (**b**) $k_{PL:HA}=1:8$; (**c**) $k_{PL:HA}=1:5$; (**d**) $k_{PL:HA}=2:7$. The legend indicates pH values.

The hydrophobic interactions analyzed in Figure 12 depict the total number of molecular interactions in the system, including both HA and DPPC molecules. The drastic increase in interactions over that of HA alone is due to the hydrophobicity contributed by DPPC as aggregate micellar forms are created. Such micelles can be oriented either with head groups toward or away from HA molecules. Because this phenomenon is mostly due to DPPC minimizing the hydrophobic contact area with water, the orientation is strongly dependent on concentration; and, as more lipids are present, micellization forces overcome HA and DPPC interactions to minimize the hydrophobic interactions in the system.

**Figure 13:**
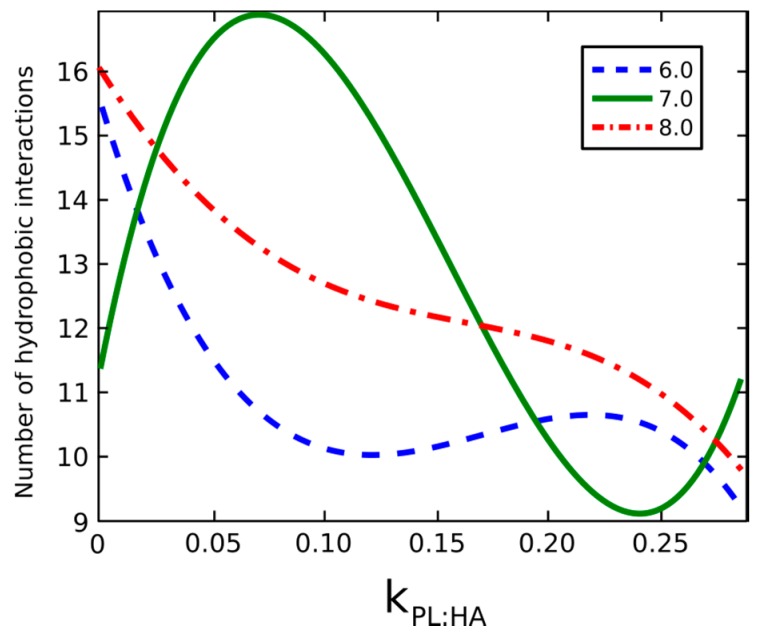
The total number of hydrophobic interactions inside HA. The legend indicates pH values.

Figure 13 presents that HA is most hydrophobic when DPPC is not present at pH = 7.0. The addition of DPPC reverses this outcome, wherein increasing concentrations of DPPC cause the hydrophobic centers of HA to be occupied. The hydrophobic tails of DPPC are directed toward HA chains, whereas the hydrophilic head groups are directed toward the solution. The number of hydrophobic interactions between HA atoms decreases. When the *R_g_* of HA decreases, hydrophilic head groups already associated with HA lipids start to interact with head groups of other already associated DPPC molecules.

When the pH value is 7, the number of hydrophobic interactions inside HA shows a maximum value at $k_{PL:HA}\approx 0.08$ and a minimum value at $k_{PL:HA}\approx 0.25$. In the absence of DPPC, hydrophobic groups of HA interact with each other hydrophobically. In the case of low DPPC concentrations, the hydrophobic tails show affinity with the hydrophobic centers of HA instead of the hydrophobic tails of other DPPC molecules. When the DPPC concentration reaches the critical mass concentration value, which is beyond the test value, DPPC begins to form micelles. Consequently, the HA hydrophobic centers are released and begin to interact with each other.

**Figure 14:**
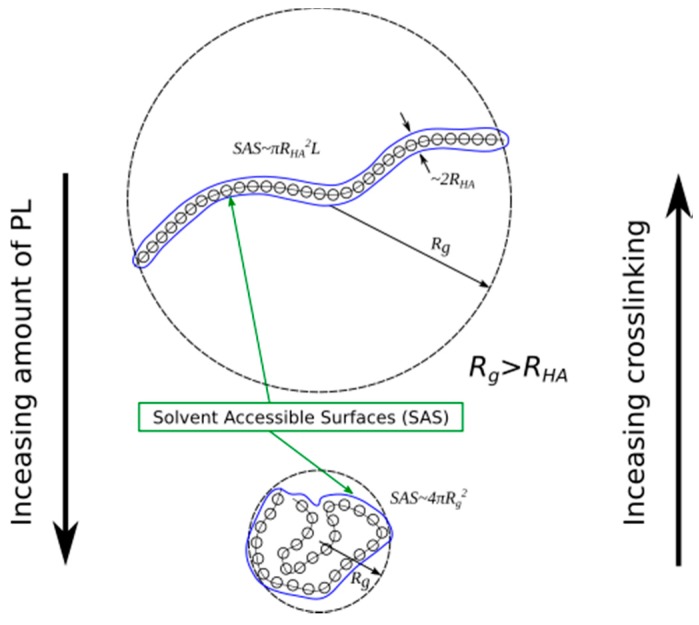
An effect of increasing PL concentration on the solvent-accessible surface and associated crosslinking ability.

An effect of increasing PL concentration on the solvent-accessible surface and the associated ability to crosslink HA is shown in Figure 14. Short HA chains in linear unfolded form are not able to produce spatial networks, since short HA chains in the presence of PL molecules tend to roll up into globular form. If the HA length is insufficient to crosslink the solvent-accessible surface of the globular form, it may be bigger than the solvent-accessible surface of HA in its linear form. Only long HA chains can crosslink through the hydrophobic centers and bridges formed by the association of HA to PL. Because of HA globalization, which in turn can be a result of an increasing concentration of PL, the viscoelastic properties of the synovial fluid worsen.

**Figure 15:**
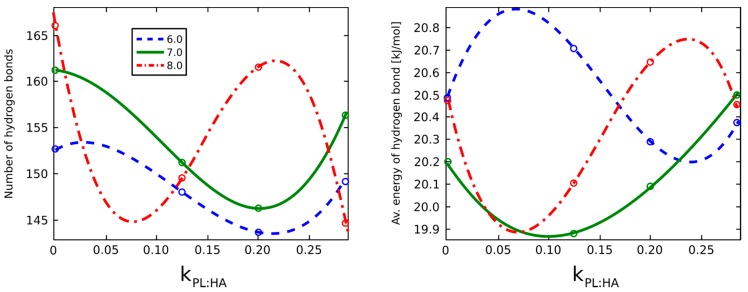
The number of hydrogen bonds (**Left**) and its average energy (**Right**) in HA as dependent on pH and PL concentration. The legend indicates pH values.

Figure 15 demonstrates the number of hydrogen bonds between the atoms of HA molecules and the respective hydrogen bond energies as dependent on pH and DPPC concentration. The number of hydrogen bonds for pH = 8.0 compared to other values is quantitatively different. The HA molecular chain size at pH = 8.0 can behave in a more random manner, a phenomenon requiring further study. The line shapes of both graphs are similar, meaning that the average binding energy is changing proportionally to the number of hydrogen bonds, which are observed between atoms of oxygen with about three to four bonds per one subunit of the HA chain.

**Figure 16:**
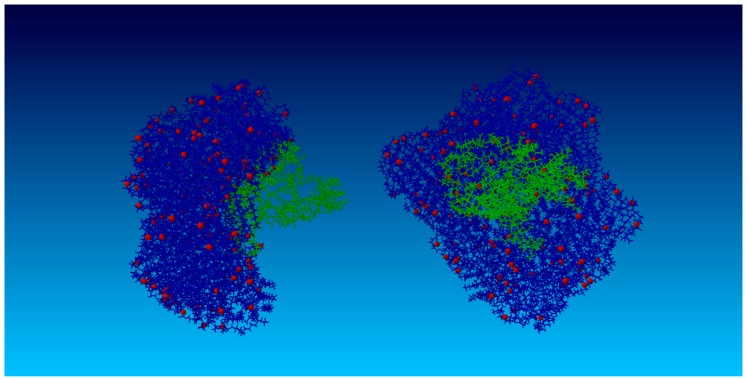
Simulation snapshot of the HA chain (**Left**) adsorbed into a DPPC vesicle (**Right**). The images are depicted with 150 lipids. HA molecules are green and DPPC molecules are blue. Red depicts oxygen atoms localized at hydrophilic DPPC head groups.

Figure 16 presents a simulation snapshot depicting the HA chain adsorbed into a DPPC vesicle. HA chains are adsorbed at the surface of the vesicle at high $k_{PL:HA}$ ratios. This mechanism is more visible, as DPPC creates a vesicle that encapsulates HA in its interior and which excludes those chains from crosslinking mechanisms.

## 3. Discussion

The relationships that exist between tribology and the mechanisms of synovial joint organ system lubrication, and hence surface degeneration, have been well-established [24,25]; and, the restoration of surface properties remains an important therapeutic goal for biotribology now that surgical advances have enabled the repair of articular cartilage beyond palliative care to a suitable substrate upon which the surface-active phospholipid layer can reconstitute [4,5,6,7]. This study begins to evaluate, as a prerequisite to tribological surgical adjuvant clinical testing, whether the lubricating molecular layer between juxtaposed articular cartilage surfaces can be normalized to further enhance tissue rescue despite osteoarthritic phenotypes that may persist after articular cartilage lesion repair and during surgical convalescence.

Based upon prior work regarding nanoscale friction in articulating systems [26,27,28,29,30,31,32,33], this study further demonstrates that the nature of HA and PL interactions plays an important role in system efficiency; and, the synovial fluid changes in pH, hyaluronic acid polydispersity, and phospholipid concentration associated with osteoarthritis, individually and collectively, contribute to overall system dysfunction. Our results indicate that effective lubrication cannot be accomplished with a preponderance of intra-articular low molecular weight HA due to its inability to crosslink into effective supramolecular structures that are necessary for facilitated lubrication. Low molecular weight HA tends toward the formation of random coils, does not interlink, and produces lower non-strain-rate-dependent viscosity. Because molecular weight influences overall synovial fluid viscosity as described by the Mark–Houwink–Sakurada law [22,34], three HA viscosity regimes undergoing Rouse and/or Zimm biopolymer chain dynamics have been described, determining that a molecular weight between 0.1 and 1.0 MDa optimizes crosslinking and network formation [34]. This network formation appears based on the longitudinal diffusion coefficient being twice the transversal counterpart, which results in the same network spring constant ratios and a molecular length dependent on the rotational diffusion coefficient [34].

Previous experimental data support our findings that HA *R_g_* is pH-dependent and its maximum *R_g_* value is observed at pH = 7 [35]. We further demonstrate that low molecular weight HA molecules exhibit linear unfolded forms which are most hydrophobic (see Figure 13), present the greatest surface accessibility (see Figure 10), maximize crosslinking affinities, and display subdiffusion behavior and greater randomness at pH = 7.0 as presented in Figure 6. Such findings would be expected under healthy synovial fluid conditions. Under osteoarthritic phenotypes, as represented by pH = 8, these features accede to a more compact folded form yielding decreased hydrophobicity, surface accessibility, and crosslinking affinities, as well as a more regular diffusion behavior and less randomness. This functional deterioration is in line with the trend toward lower molecular weight HA forms being associated with osteoarthritic synovial fluid, forms which have not been shown to play a significant role in facilitated lubrication regimes [13,19,20]. Nonetheless, HA crosslinking mechanisms provide carrier functions, enable wetting, and maintain superficial active phospholipid layers important for lubrication [36,37,38,39,40], and so crosslinking should remain an adjuvant design parameter.

In the presence of DPPC molecules, our data indicate a time delay in HA *R_g_* stabilization, reflecting the kinetic folding rate and less random behavior governed by the hydrophobic interactions between molecular species. A progressive decrease in *R_g_* with an increasing DPPC (Figure 3 and Figure 4) concentration for pH = 7 was observed, while pH = 8 displayed irregular *R_g_* contraction features over time. DPPC decreased HA hydrophobicity, as increasing DPPC concentrations caused the HA hydrophobic centers to be occupied such that the hydrophobic tails of DPPC are visualized as being directed toward HA chains, whereas hydrophilic head groups toward the solution with the number of hydrophobic interactions between HA atoms decreased. When HA *R_g_* decreases, the hydrophilic head groups already associated with HA begin to interact with head groups of other already associated DPPC molecules. Short HA chains displaying linear unfolded forms tend to roll up into globular forms which tend to associate with PL, as HA hydrophobic sites are available and consequently unable to organize into large supramolecular PL–HA spatial networks. On the other hand, longer chains adsorb PL, causing *R_g_* decreases which themselves can prevent contribution to network formation. Only large HA chains exhibit the simultaneous capability to crosslink through intra- and inter-chain hydrophobic centers and to absorb to PL head groups that form surface-active complexes. Such hydrophobic and electrostatic phenomena are notable with DPPC [25,41]. An additional consequence of HA globalization secondary to increasing PL concentration is the worsening of synovial fluid viscoelastic properties [42], further reflecting system dysfunction.

Our data indicated that the average binding energy changed proportionally to the number of hydrogen bonds and HA chains were adsorbed at vesicle surfaces at high $k_{PL:HA}$ ratios (Figure 15). This mechanism was even more pronounced, as DPPC created a vesicle encapsulating HA in its interior and excluded those chains from crosslinking opportunities [43]. At pH = 7.0, no significant surface area changes were observed among DPPC concentrations when compared to other pH values that exhibit a variable surface area. Because this phenomenon is mostly due to DPPC minimizing the hydrophobic contact area with water, the orientation was strongly dependent on concentration; and as more DPPC molecules were present, micellization forces overcame HA and DPPC interactions to minimize the hydrophobic interactions in the system.

The PL increase in osteoarthritic synovial fluid results in a higher number of aggregated forms, such as micelles and reverse micelles, which may not provide a proper response to certain external loads due to the lack of HA networks preventing micellar collapse. Significantly, mechanical damage to the continuity of articular cartilage surfaces can be manifest as an increased synovial fluid pH and PL molecule concentration without significantly affecting *R_g_*. In aqueous solutions such as synovial fluid, non-polar groups of atoms tend to aggregate, since aggregation reduces the number of water molecules that must participate in an ordered, and entropically unfavorable, interfacial cage structure. This water-mediated hydrophobic effect can be interpreted as an attractive hydrophobic interaction between nonpolar groups of atoms. Statistically, hydrophobic interactions are rather long-ranged, extending up to 7 Å, such that a space deficiency for water molecules exists between the interacting groups, leading to an unfavorable vacuum and thus strong attractive forces.

PL composition, including molecular saturation levels and fatty acid chain length, is significantly transformed during osteoarthritic pathogenesis. Although unsaturated, rather than saturated, phosphatidylcholines have been linked to the lubrication of synovial joint organ systems [14,15], DPPC should not yet be abandoned as an adjuvant candidate in certain clinical situations. Like sphingomyelin [29,44,45], whose hydrophobic chains are highly saturated, prone to intermolecular hydrogen bonding and lipid raft formation, and exhibit a higher phase transition temperature, DPPC may be important for mitigating heat exchanges associated with the deformation cycles of diseased articular surfaces due to its own higher gel–liquid crystal transition temperature [15,29,46,47]. Because PLs are present on articular cartilage surfaces in layer and vesicle form, phase transition temperature may be important, in addition to lateral layer heterogeneity, for self-assembly capabilities in association with HA through perturbation forces typically encountered post-treatment [29,48]. HA associates with the outer shell of DPPC in vesicle form in bulk solution, which can be deposited on to surfaces—easily deformed into other structures, such as multilayers—or removed altogether under higher loads. Further, it has been recognized that when PL progresses deeper into its solid phase, better lubrication is often created, although some PLs in liquid disordered phases may be preferable simply based upon anticipated loading conditions [49,50]. Such post-operative loading after cartilage repair is a complex variable requiring further assessment [51].

We remain optimistic that tribological surgical adjuvants can be deployed toward improving chemomechanotransductive environments at articular cartilage repair sites sufficient to warrant wear mitigation studies. In this work, we investigated how the small-sized HA faction behaves with increasing pH values and PL concentration as observed in osteoarthritis. While we have demonstrated that low molecular weight HA, by interacting with PL, would be ineffective for facilitated lubrication and deteriorates synovial fluid viscoelastic properties by nonparticipation in the crosslinking of long-chain HA molecules, DPPC may play a useful adjuvant role in certain clinical situations. An additional evaluation of HA and PL interactions is required to determine in vivo molecular benchmarks that would facilitate optimal absorption–desorption kinetics at surgically repaired surfaces toward re-establishing an effective lubricating layer [52] to enhance tissue rescue. The framework deployed in this study, namely to evaluate phenotypic changes that occur commensurate with the disease state, can be used to optimize repair site durability and to estimate the point-of-no-return at which articular cartilage lesions should remain relegated to palliative care.

## 4. Materials and Methods

### 4.1. The Simulation Setup

**Figure 17:**
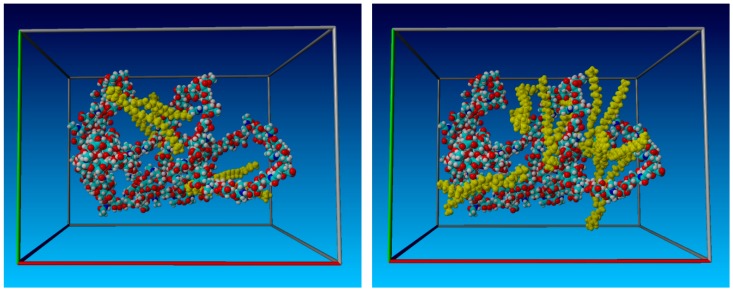
The initial simulation box configuration containing a single HA chain and three (**Left**) and seven (**Right**) particles of the DPPC. The HA chain is partially folded to reduce the total number of atoms; and, for better visualization, the water molecules are not shown. DPPC particles are yellow.

Figure 17 illustrates the initial simulation box configuration. Because the low molecular weight HA chain is relatively long (~50 nm), it has been partially folded into a more compact form. Otherwise, the size of the simulation box must be much greater. For larger simulation boxes, the total number of atoms, including water molecules, is too large for a personal computer (PC)-class computer. For the presented configuration, the volume of the box is about 300 nm^3^ (8.4 nm × 6.2 nm × 5.8 nm).

Throughout the simulation, the total number of atoms remained constant. The simulation began with a random distribution of HA and DPPC molecules in the simulation box to avoid overlapping. The box was subsequently filled with water containing 0.9% NaCl in the amount corresponding to established water density. With a random distribution of the molecules in the simulation box, the distance between atoms and molecules can be much less than the distance at which the interparticle potential is zero (see *σ* in the Lennard–Jones potential). To avoid an explosion of simulation box energy, minimalization is required. YASARA performs this task automatically by shifting the colliding and overlapping atoms. The pressure and temperature remained constant at 1 bar and 310 K (37 °C), respectively. The total number of atoms depended on the amount of DPPC particles (three, five, or seven) and the pH of the solution; see Section 4.3 below.

### 4.2. Force Field

An Assisted Model Building with Energy Refinement (AMBER) force field has been employed to mimic the interactions between molecules. The functional form of the AMBER03 force field is:

(1) $$E_{total}=E_{bonds}+E_{angles}+E_{dihedrals}+E_{vaW}+E_{electrostatic},$$

where *E_bonds_* represents the energy between covalently bonded atoms, *E_angles_* represents the energy due to the geometry of electron orbitals involved in covalent bonding, *E_dihedrals_* represents the energy for twisting a bond due to bond order (e.g., double bonds) and neighboring bonds or lone pairs of electrons, *E_vdW_* represents the non-bonded energy between all atom pairs (van der Waals energy), and *E_electrostatic_* represents the electrostatic interactions between all atom pairs. Further, deploying the YASARA Structure package, molecular dynamics simulations were performed to study PL–HA interactions. The methods for surface area calculation follow that previously utilized [53,54].

### 4.3. pH Setting and Control

In YASARA, the pH setting only affects the protonation states of organic molecules, not the composition of the solvent. A program database (PDB) files provide only basic connectivity information for heteroatoms at a rather low resolution and without hydrogen atoms. The knowledge about the order of covalent bonds is crucial for adding hydrogen atoms or automatically assigning force field parameters. YASARA customarily assigns bond orders and protonation patterns according to pH 7.0. In changing pH values, YASARA reassigns the order of all bonds and subsequently adds the missing hydrogen atoms to match the new pH value (for more details on the automatic assigning of parameters see the YASARA documentation).

### 4.4. Radius of Gyration

The root-mean-square deviation (RMSD) between the Cartesian atom coordinates in two selections is represented according to the following formula, where *R* is the vector linking the *n* corresponding atom pairs in space:

(2) $$\sigma_{R}=\sqrt{\frac{\sum_{i=1}^{n}R_{i}\cdot R_{i}}{n}}.$$

*R_g_* is defined as the root-mean-square distance of the atoms from the center and can be calculated twofold. First, if the center is assigned to the geometric center of a molecule, then

(3) $$R_{g\left(geometric\right)}=\sqrt{\frac{1}{N}\sum_{i=1}^{N}{\left(R_{i}-C\right)}^{2}},$$

where *R_i_* is a position of the *i*-th atom and *C* is position of the geometrical center of the molecule. Second, when the center is assigned to the center of mass, *R_g_* is defined as

(4) $$R_{g\left(mass\right)}=\sqrt{\frac{\sum_{I=1}^{n}M_{i}{\left(R_{i}-C\right)}^{2}}{\sum_{i=1}^{N}M_{i}}},$$

where *M_i_* is a mass of the *i*th atom. In this study, *R_g(geometric)_* was analyzed.

### 4.5. Rouse Model Application

**Figure 18:**
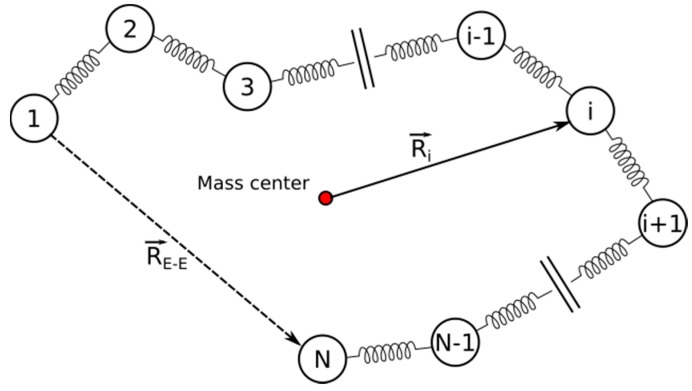
Rouse bead-spring polymer model.

The Rouse model is used to analyze HA at different conditions. The model is widely used in polymer physics and describes the conformational dynamics of an ideal chain. Single chain movement is represented as Brownian motion of beads connected by harmonic springs. There are no excluded volume interactions between the beads, and each bead is subjected to random thermal and drag forces as in Langevin dynamics [55,56]. Figure 18 presents a generic example of the Rouse model in which a bead-spring polymer is depicted.

The parameters analyzed include end-to-end vectors represented as

(5) $$\vec{R}=\sum_{i}\vec{r_{i}},$$

the end mean square displacement of center of mass represented as

(6) $$<{\left(R_{G}\left(t\right)-R_{G}\left(0\right)\right)}^{2}>=\sum_{a=x,y,z}<{\left(X_{\alpha }\left(t\right)-X_{\alpha }\left(0\right)\right)}^{2}>=6\frac{k_{\beta }T}{N\xi }t,$$

and an autocorrelation function of the end-to-end vector determined by

(7) $$\langle R\left(t\right)\cdot R\left(0\right)\rangle ≅\sum_{i:odd}\frac{1}{i^{2}}\exp\left(-\frac{t}{\tau_{i}}\right).$$

Formula (6) is simplified to the following form

(8) $$\langle R\left(t\right)\cdot R\left(0\right)\rangle ≅Nb^{2}\exp\left(-\frac{t}{\tau_{1}}\right),$$

where *N* is the number of beads, *τ* is the relaxation time, and *b* is the length of a string.

### 4.6. Hydrophobic Interactions

**Figure 19:**
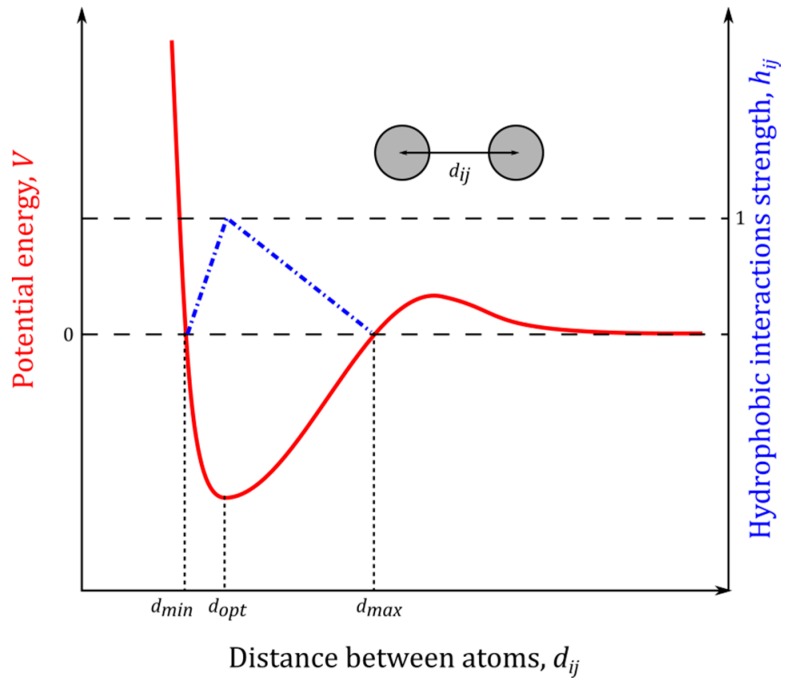
An example of the simplified knowledge-based potential extracted from the high-resolution X-ray structure in the PDB files. The interaction range consists of $d_{min}$, $d_{opt}$, and $d_{max}$ as shown.

The hydrophobic interaction strength between hydrophobic atoms (Figure 19) is calculated by the following algorithm. Hydrophobic atoms are identified and assigned an atom type from the groups depicted as type 1 for carbon atoms with three or more bound hydrogen atoms (–CH3), type 2 for carbon atoms with two hydrogen atoms or one hydrogen atom plus three carbon atoms bound (–CH2–, HCC3), and type 3 for aromatic ring carbon atoms with only carbon and hydrogen atoms bound. For each of the resulting six hydrophobic interactions, a knowledge-based potential was extracted from high-resolution X-ray structures in the PDB files, and the favorable interaction range was determined as per YASARA. This range consists of $d_{min}$ as the minimum distance where the energy becomes positive due to clashes, $d_{opt}$ as the optimum distance equating to the energy minimum, and $d_{max}$ as the maximum distance where energies become positive due to the cost of creating vacuum between the atoms.

The values of $d_{min}$,$\;d_{opt}$ and $d_{max}$ for all types of interacting hydrophobic atoms are provided in Table 3.

Individual contribution, $h_{ij}$, is interatomic distance, $d_{ij}$, dependent as follows:

(9) $$h_{ij}=\{\begin{array}{c} \mathrm{if}\;d_{ij}\in \left(d_{min};d_{opt}\right):\;h_{ij}=\frac{d_{ij}-d_{min}}{d_{opt}-d_{min}}\;\\ \mathrm{if}\;d_{ij}=d_{opt}:\;h_{ij}=1\\ \mathrm{if}\;d_{ij}\in \left(d_{opt};d_{max}\right):\;h_{ij}=\frac{d_{max}-d_{ij}}{d_{max}-d_{opt}}\; \end{array}.$$

Each hydrophobic atom pair thus contributes to the total interaction strength with a value in the range from 0 (for $d_{ij}=d_{min}$ and $d_{ij}=d_{max}$) to 1 (for $d_{ij}=d_{opt}$). The total hydrophobic interaction strength, $H_{total}$, is determined by the sum of the contributions from all hydrophobic atom pairs:

(10) $$H_{total}=\sum_{i\neq j}h_{ij}.$$

In YASARA, hydrophobic interaction strength is normalized for each hydrophobic atom–atom interaction such that it cannot be treated as a classical Newton force. Interaction strengths with an $H_{total}$ above 5.0 indicate strongly interacting residues.

### 4.7. Hydrogen Bond Identification and Strength

YASARA’s definition of a hydrogen bond (HB) is that the hydrogen bond energy is greater than 6.25 kJ/mol (or 1.5 kcal/mol), which is 25% of the optimum value 25 kJ/mol. The following formula yields the bond energy in kJ/mol as a function of the Hydrogen-Acceptor distance and two scaling factors:

(11) $$E_{HB}=25\cdot \frac{2.6-\max\left(Dis_{H-A,}2.1\right)}{0.5}\cdot Scale_{D-A-H}\cdot Scale_{H-A-X},$$

where in the first scaling factor depends on the angle formed by Donor-Hydrogen-Acceptor, and the second scaling factor is derived from the angle formed by Hydrogen-Acceptor-X, where the latter *X* is the atom covalently bound to the acceptor. Both scaling factors vary from 0 to 1 as follows:

(12) $$Scale_{H-A-X}\{\begin{array}{c} 0\\ 0\ldots 1\\ 1 \end{array}\begin{array}{c} \mathrm{in}\;\mathrm{range}\;0\ldots 100\;\mathrm{degrees}\\ \mathrm{in}\;\mathrm{range}\;100\ldots 165\;\mathrm{degrees}\\ \mathrm{in}\;\mathrm{range}\;165\ldots 180\;\mathrm{degrees} \end{array},$$

if *X* is a heavy atom, the second scaling factor is

(13) $$Scale_{H-A-X}\{\begin{array}{c} 0\\ 0\ldots 1\\ 1 \end{array}\begin{array}{c} \mathrm{in}\;\mathrm{range}\;0\ldots 85\;\mathrm{degrees}\\ \mathrm{in}\;\mathrm{range}\;85\ldots 95\;\mathrm{degrees}\\ \mathrm{in}\;\mathrm{range}\;95\ldots 180\;\mathrm{degrees} \end{array},$$

if *X* is a hydrogen, slightly smaller angles are allowed, and the scaling factor then is

(14) $$Scale_{H-A-X}\{\begin{array}{c} 0\\ 0\ldots 1\\ 1 \end{array}\begin{array}{c} \mathrm{in}\;\mathrm{range}\;0\ldots 75\;\mathrm{degrees}\\ \mathrm{in}\;\mathrm{range}\;75\ldots 85\;\mathrm{degrees}\\ \mathrm{in}\;\mathrm{range}\;85\ldots 180\;\mathrm{degrees} \end{array},$$

If the Acceptor forms more than one covalent bond, the one with the lowest scaling factor is taken.

### 4.8. Other Simulations

**Figure 20:**
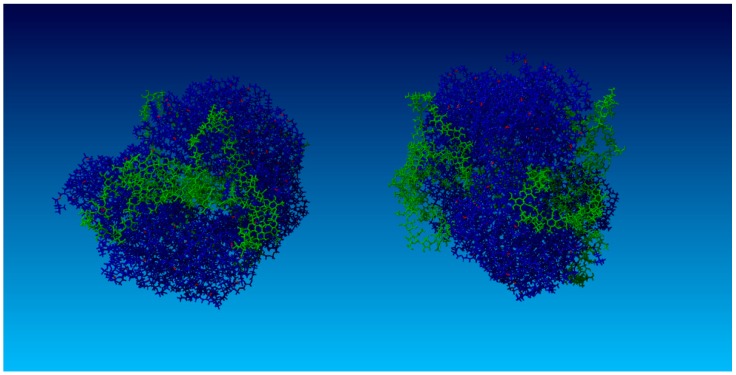
Simulation snapshot of the 192 nm HA chain (**Left**) adsorbed into a DPPC vesicle (**Right**). HA molecules are green and DPPC molecules are blue. Red depicts oxygen atoms localized at hydrophilic DPPC head groups.

As an additional study design validation, 192 nm HA (80 kDa) was tested. Similar behavior was observed, consistent with the findings that HA < 0.5 MDa is absorbed by PL micelles [43]; see Figure 20.

### 4.9. Limitations

A limitation of this study is that PDB files often contain ligands and cofactors at a rather low resolution and without hydrogen atoms, which create challenges for a computer program to automatically analyze the molecule, assign the right bond orders, and subsequently add the missing hydrogen atoms. Additionally, difficulties arise from the fact that bond orders and protonation states depend on the pH to be simulated. YASARA normally assigns bond orders and protonation patterns according to pH 7.0, and the pH is controlled through reassigning bond orders or adding missing hydrogen atoms as described in Section 4.3 above.

## Figures and Tables

**Table 1:** Gaussian function fitting parameters for Figure 4. *R*^2^ is the coefficient of multiple determinations for regression, which ranges from 0 to 1 and measures how close the data are to the fitted regression and how well the observed outcomes are replicated by the model. μ is an expected value, also known as the expectation, average, mean value, or first moment; and, $\sigma^{2}$ is an expectation variance value of the squared deviation of a random variable from its mean, also informally measuring how far a set of numbers are spread from their mean.

pH	*R*^2^	μ	$\sigma^{2}$
6.0	0.9661	0.2021	0.152
7.0	1.0000	0.0230	0.165
8.0	0.9964	0.1756	0.217

**Table 2:** Individual goodness-of-fit and exponents of the mean square displacement. *R*^2^ is the coefficient of multiple determinations for regression, which ranges from 0 to 1 and measures how close the data are to the fitted regression line and how well the observed outcomes are replicated by the model.

pH	*R*^2^	*β*
6.0	0.9493	0.6167
7.0	0.9969	0.6744
8.0	0.9834	1.0932

**Table 3:** The minimum, optimal, and maximum distances between interacting hydrophobic atoms utilized to determine interaction strength.

Atom Types	Type 1 (–CH3)	Type 2 (–CH2–; HCC3)	Type 3 (Aromatic)
Type 1 (–CH3)	$dis_{min}=3.50\;Å$ $dis_{opt}=4.00\;Å$ $dis_{max}=6.78\;Å$	$dis_{min}=3.68\;Å$ $dis_{opt}=4.20\;Å$ $dis_{max}=6.70\;Å$	$dis_{min}=3.52\;Å$ $dis_{opt}=4.05\;Å$ $dis_{max}=7.00\;Å$
Type 2 (–CH2–; HCC3)		$dis_{min}=3.63\;Å$ $dis_{opt}=4.11\;Å$ $dis_{max}=6.62\;Å$	$dis_{min}=3.48\;Å$ $dis_{opt}=4.07\;Å$ $dis_{max}=6.64\;Å$
Type 3 (aromatic)			$dis_{min}=3.39\;Å$ $dis_{opt}=3.91\;Å$ $dis_{max}=6.81\;Å$
